# P2X7 Receptor Activation Modulates Autophagy in SOD1-G93A Mouse Microglia

**DOI:** 10.3389/fncel.2017.00249

**Published:** 2017-08-21

**Authors:** Paola Fabbrizio, Susanna Amadio, Savina Apolloni, Cinzia Volonté

**Affiliations:** ^1^IRCCS Santa Lucia Foundation, Experimental Neuroscience Rome, Italy; ^2^Department of Systems Medicine, Tor Vergata University Rome, Italy; ^3^CNR, Institute of Cell Biology and Neurobiology Rome, Italy

**Keywords:** ALS, ATP, BzATP, LC3B-II, microglia, P2X7

## Abstract

Autophagy and inflammation play determinant roles in the pathogenesis of Amyotrophic Lateral Sclerosis (ALS), an adult-onset neurodegenerative disease characterized by deterioration and final loss of upper and lower motor neurons (MN) priming microglia to sustain neuroinflammation and a vicious cycle of neurodegeneration. Given that extracellular ATP through P2X7 receptor constitutes a neuron-to-microglia alarm signal implicated in ALS, and that P2X7 affects autophagy in immune cells, we have investigated if autophagy can be directly triggered by P2X7 activation in primary microglia from superoxide dismutase 1 (SOD1)-G93A mice. We report that P2X7 enhances the expression of the autophagic marker microtubule-associated protein 1 light chain 3 (LC3)-II, via mTOR pathway and concomitantly with modulation of anti-inflammatory M2 microglia markers. We also demonstrate that the autophagic target SQSTM1/p62 is decreased in SOD1-G93A microglia after a short stimulation of P2X7, but increased after a sustained challenge. These effects are prevented by the P2X7 antagonist A-804598, and the autophagy/phosphoinositide-3-kinase inhibitor wortmannin (WM). Finally, a chronic *in vivo* treatment with A-804598 in SOD1-G93A mice decreases the expression of SQSTM1/p62 in lumbar spinal cord at end stage of disease. These data identify the modulation of the autophagic flux as a novel mechanism by which P2X7 activates ALS-microglia, to be considered for further investigations in ALS.

## Introduction

Neuroinflammation plays a determinant role in the pathogenesis of Amyotrophic Lateral Sclerosis (ALS; Cozzolino et al., 2012), where degenerating motor neurons (MN) contribute to produce danger signals that activate microglia to initiate a self-propagating cytotoxic cascade resulting in muscle weakness, atrophy, spasticity, compromised breathing and finally death in patients (Henkel et al., 2009; Ilieva et al., 2009). Extracellular ATP functions as one of such endogenous alarm signal, by operating through purinergic P2 receptors, particularly the P2X7 ionotropic subtype (Beamer et al., 2016; Illes and Verkhratsky, 2016). Activation of P2X7 in microglia purified from superoxide dismutase 1 (SOD1)-G93A mouse model of ALS exacerbates pro-inflammatory responses exemplified by the levels of cyclooxygenase-2 (COX-2), mitogen-activated protein kinase (MAPK), NADPH oxidase 2 (NOX2), nuclear factor kappa-B (NF-κB), tumor necrosis factor alpha (TNF-α), with a consequent cytotoxicity towards neurons (D’Ambrosi et al., 2009; Apolloni et al., 2013b; Parisi et al., 2016). In the SOD1-G93A mouse model of ALS, we have previously reported that disease onset is significantly anticipated and disease progression is worsened in mice genetically lacking P2X7, thus suggesting that the receptor has beneficial effects at least during a precise stage of the disease that is however still undefined (Apolloni et al., 2013a). Only when administered to SOD1-G93A mice from late pre-onset, the P2X7 antagonist Brilliant Blue G enhances MN survival and reduces microgliosis in lumbar spinal cord (Apolloni et al., 2014), with a slight beneficial effect on disease progression and mice survival (Cervetto et al., 2013; Apolloni et al., 2014; Bartlett et al., 2017). By confirming the dual role previously recognized for P2X7 (Monif et al., 2010, 2016), these findings have highlighted how crucial is the lag of P2X7 modulation for eliciting beneficial effects in ALS.

Autophagy is an ubiquitous and highly conserved homeostatic mechanism by which eukaryotic cells degrade damaged organelles, intracellular components and protein aggregates. It consists in the sequestration of target cellular materials (cargo) into autophagosomes and their subsequent transfer to and catabolism by the autophagosome-lysosome (autolysosome vacuole) system (Klionsky and Emr, 2000; Glick et al., 2010). Abnormalities in autophagy have been implicated in chronic neurodegenerative conditions comprising ALS, where both patients and animal models are characterized by overexpression of the autophagy marker microtubule-associated protein 1 light chain 3 (LC3)-II in spinal cord (Song et al., 2012). Moreover, sequestosome-1, a protein also known as ubiquitin-binding protein SQSTM1/p62 encoded by the SQSTM1 gene, which is an autophagosome receptor recruiting proteins for selective autophagy, is found accumulated in spinal cord of SOD1-G93A mice, concomitantly with increased extent of autophagic vacuoles (Zhang et al., 2011).

In mouse microglia and in human epithelial cells, P2X7 has been reported to negatively regulate autophagy by impairing lysosomal function (Takenouchi et al., 2009a; Haanes et al., 2012). Conversely, P2X7 apparently increases autophagy in monocytes and macrophages during mycobacterial infections (Biswas et al., 2008), and acts as a positive regulator of autophagy in dystrophic muscle cells (Young et al., 2015). This not only confirms the twofold role often played by P2X7, but also highlights a context-specific effect of P2X7 on autophagy pathways.

The purpose of this work is thus to learn if P2X7 might participate to ALS pathogenesis by directly modulating autophagy, and particularly the expression of the autophagosome component LC3-II and of the autophagy receptor p62 in SOD1-G93A microglia. We demonstrate that activation of P2X7 deregulates LC3-II and p62 protein content. Moreover, *in vivo* treatment with the specific P2X7 antagonist A-804598 reduces p62 levels in lumbar spinal cord of SOD1-G93A mice at end stage of disease. These data identify the modulation of autophagy as a novel mechanism by which P2X7 activates ALS-microglia.

## Materials and Methods

### Reagents

ATP, 2′-3′-O-(benzoyl-benzoyl) ATP (BzATP) and all other reagents, unless otherwise stated, were obtained from Sigma Aldrich (Milan, Italy). PD98059 was purchased from Calbiochem (San Diego, CA, USA). A-839977 and A-804598 were from Tocris Bioscience (Bristol, UK).

### Antibodies

Arginase-1 (ARG-1) rabbit antibody (1:700) was from Abcam (Cambridge, UK); CD163 rabbit antibody (1:100) was from Santa Cruz Biotechnology, Inc. (Dallas, TX, USA); LC3B antibody (1:500); mTOR (1:500) and p-mTOR (1:500) rabbit antibodies were from Cell Signaling Technology Inc. (Beverly, MA, USA). SQSTM1/p62 mouse monoclonal antibody (1:500) was obtained from Abcam (Cambridge, UK). GAPDH mouse antibody (1:2500) from Calbiochem (San Diego, CA, USA) was used for protein normalization. HRP-linked anti-rabbit and anti-mouse antibodies were from Jackson Immunoresearch (West Grove, PA, USA).

### Mice

All animal procedures have been performed according to the European Guidelines for the use of animals in research (86/609/CEE) and the requirements of Italian laws (D.L. 26/2014). The ethical procedure has been approved by the Animal Welfare Office, Department of Public Health and Veterinary, Nutrition and Food Safety, General Management of Animal Care and Veterinary Drugs of the Italian Ministry of Health (protocol number 319/2015PR). All efforts were made to minimize animal suffering and use the minimum number of animals necessary to obtain reliable results. Adult B6.Cg-Tg(SOD1-G93A)1Gur/J mice were obtained from Jackson Laboratories (Bar Harbor, ME, USA), bred in our indoor animal facility and genotyped as described (Apolloni et al., 2013a). To determine behavioral scores we employed a scale from 5 to 1, where 5 defines healthy mice without symptoms of paralysis, 4 indicates mice with slight signs of destabilized gait and paralysis of the hind limbs, 3 describes mice with clear paralysis and destabilized gait, 2 depicts mice with fully developed paralysis of the hind limbs that only crawl on the forelimbs, finally 1 defines mice with fully developed paralysis of the hind limbs that predominantly lie on the side and/or are not able to straighten up within 30 s when they are turned on their back. After reaching a score of 1 (end stage, ~23 weeks of age), the animals were euthanized, according to the guidelines for preclinical testing and colony management (Ludolph et al., 2010). SOD1-G93A mice were considered at onset when exhibited a statistically significant 10% decline of rotarod performance, with respect to wild-type (WT) mice. Rotarod performance was evaluated by a rotarod apparatus (Ugo Basile 7650 model) at a constant speed of 15 r.p.m. over a maximum period of 180 s (Apolloni et al., 2013a).

SOD1-G93A mice at 100 days of age (pre-onset) were randomly grouped into vehicle-treated or CNS penetrant P2X7 specific antagonist A-804598-treated mice (Donnelly-Roberts et al., 2009; Catanzaro et al., 2014; Iwata et al., 2016) given by intraperitoneal injection at 30 mg/kg five times a week until end stage of disease. Because there is sex diversity in response to pharmacological treatments (Pizzasegola et al., 2009) and the P2X7 antagonist Brilliant Blue G has prolonged survival only in female SOD1-G93A mice (Bartlett et al., 2017; Sluyter et al., 2017), we have chosen to study female mice.

### Primary Microglia Cultures

Primary microglia cultures from brain cortex were prepared as previously described (Apolloni et al., 2013b). Briefly, neonatal SOD1-G93A mice and their non-transgenic littermates mice were sacrificed and, after removing the meninges, cortices were minced and digested with 0.25% trypsin and 0.01% DNaseI. After dissociation and passage through 70 μm filters, cells were resuspended in DMEM/F-12 media with GlutaMAX™ (Gibco, Invitrogen, UK), plus 10% fetal bovine serum (FBS), 100 Units/ml gentamicine and 100 μg/ml streptomycin/penicillin. After approximately 15 days, a mild trypsinization was done for 30 min at 37°C. The resultant adherent microglial cells (pure >99%) were washed twice with DMEM/F-12 and kept at 37°C in a 5% CO_2_ and 95% air atmosphere and used for experiments at least 48 h after.

### Western Blotting

Microglia in serum-free medium were harvested in SDS Laemmli sample buffer. Protein lysates were obtained by homogenization of mice lumbar spinal cords segments in homogenization buffer as described (Apolloni et al., 2014). Analysis of protein components (10 μg for tissue extracts) was performed by Mini-PROTEAN^®^ TGX™ Gels (BioRad, USA) and transfer onto nitrocellulose membranes (Amersham Biosciences, USA). After saturation with blocking agent, blots were incubated overnight at 4°C with the specified antibody, then for 1 h with HRP-conjugated secondary antibodies and visualized using ECL Advance Western blot detection kit (Amersham Biosciences, USA). Signal intensity quantification was performed by Kodak Image Station analysis software.

### Immunofluorescence and Confocal Microscopy

Primary microglia were fixed for 20 min in 4% paraformaldehyde, permeabilized in PBS containing 0.1% Triton X-100 and then incubated for 1.5 h at 37°C with anti-LC3B (1:200), anti-SQSTM1/p62 (1:100) or anti-CD11b (1:200, AbD Serotec, USA) in 1% BSA in PBS. Cells were stained for 1 h with Cy2-conjugated donkey anti-rabbit IgG or Cy3-conjugated donkey anti-mouse IgG or Cy5-conjugated donkey anti-rat IgG (1:200, Jackson Immunoresearch). Höechst 33342 was used for nuclei staining (1:1000) in PBS. Immunofluorescence was analyzed by means of a confocal laser scanning microscope (Zeiss, LSM700, Germany) equipped with four laser lines: 405 nm, 488 nm, 561 nm and 639 nm. The brightness and contrast of the digital images were adjusted using the Zen software.

### Data Analysis

Data are presented as mean ± standard error of the mean (SEM). Statistical differences were verified by student’s *t*-test if the normality test was passed, or by the Mann–Whitney rank sum test, if the normality test failed, considering acceptable a value for asymmetry between −2 and +2 in order to prove normal univariate distribution. One-way analysis of variance (ANOVA) followed by *Post Hoc* Tukey’s test was used for multiple comparisons. For onset and survival data, differences in the values of A804598- and vehicle-treated mice were assessed using Kaplan-Meier analysis paired with log-rank tests. The software package GraphPad Prism 7.03 (GraphPad Software, San Diego, CA, USA) was used for all statistical analysis with differences considered significant for *p* < 0.05.

## Results

### P2X7 Activation Induces LC3B-II Formation in SOD1-G93A Primary Microglia

LC3-II localizing to the autophagosome membrane is widely used to monitor autophagy. In particular, the generation of LC3-II from LC3-I is generally identified by immunoblot analysis, because the amount of LC3-II is strictly correlated with the number of autophagosomes and is considered a more reliable marker than LC3-I to be detected (Mizushima and Yoshimori, 2007; Barth et al., 2010; Klionsky et al., 2016). To directly investigate the modulation of the autophagic pathway by P2X7 activation in SOD1-G93A microglia, we thus compared the expression of LC3B-II, with respect to GAPDH.

**Figure 1:**
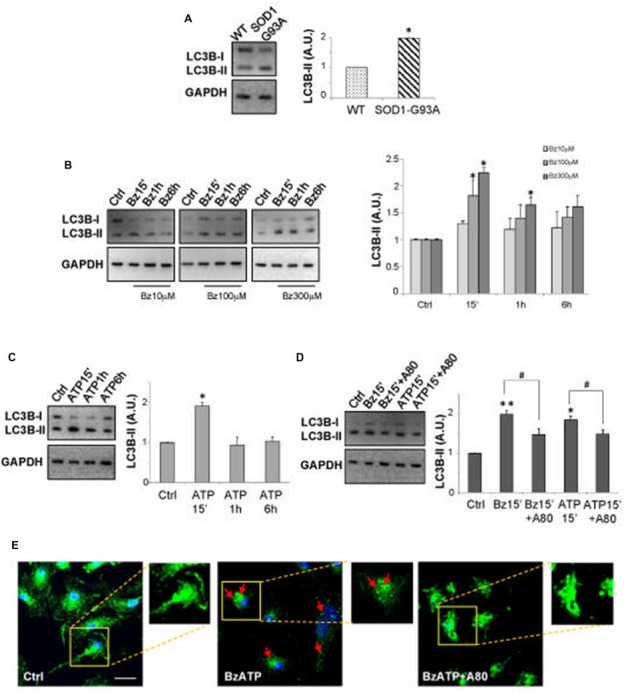
P2X7 activation modulates microtubule-associated protein 1 light chain 3 (LC3)-II in superoxide dismutase 1 (SOD1)-G93A microglia. **(A)** Equal amounts of total protein from wild-type (WT) and SOD1-G93A primary microglia were subjected to western blotting and immunoreactions with anti-LC3B-II and anti-GAPDH to normalize proteins. **(B–D)** Equal amounts of total protein from SOD1-G93A primary microglia were subjected to western blotting and immunoreactions with anti-LC3B-II and anti-GAPDH. In **(B)**, cells were exposed to 10–100–300 μM 2′-3′-O-(benzoyl-benzoyl) ATP (BzATP) for 15 min, 1 h or 6 h. In **(C)** cells were exposed to 3 mM ATP for 15 min, 1 h or 6 h. Cells were treated for 15 min with 300 μM BzATP or 3 mM ATP, in the absence or presence of 10 μM A-804598 **(D)**. **(E)** SOD1-G93A microglia treated with 300 μM BzATP in the absence or presence of 10 μM A-804598 were analyzed by means of fluorescence microscopy after staining with anti-LC3B (arrows indicate LC3 vacuoles; scale bar 50 μm; nuclei in blue). Data represent mean ± SEM of *n* = 3 independent experiments. Statistical significance was calculated by student’s *t*-test or Mann-Whitney rank sum test or analysis of variance (ANOVA) followed by *Post Hoc* Tukey’s test, and referred to WT **(A)** or Ctrl **(B–D)** (*p < 0.05, **p < 0.01); to BzATP15’-treated cells or ATP15’-treated cells (^#^*p* < 0.05).

**Figure 2:**
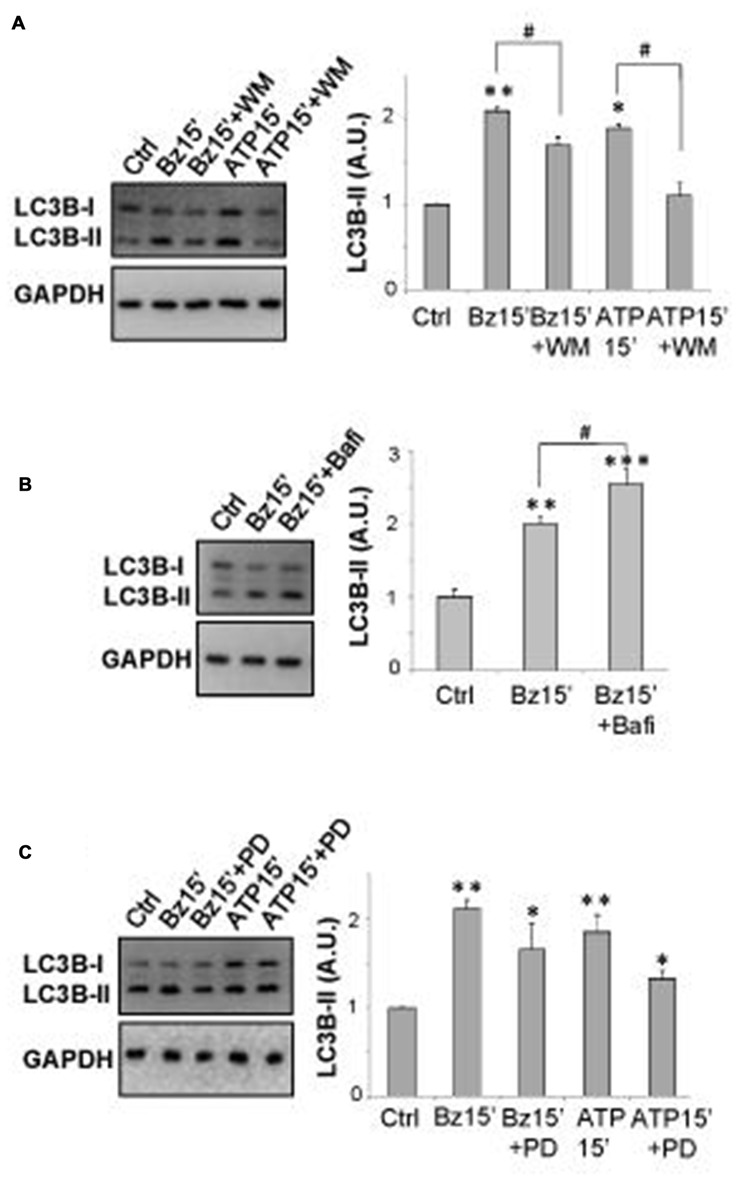
P2X7 induces autophagic flux in SOD1-G93A microglia. Equal amounts of protein from SOD1-G93A primary microglia were subjected to western blotting and immunoreactions with anti-LC3B-II and anti-GAPDH. In **(A)** SOD1-G93A microglia were treated for 15 min with 300 μM BzATP or 3 mM ATP in the absence or presence of 100 nM wortmannin (WM) **(A)**. In **(B)** SOD1-G93A microglia were treated for 15 min with 300 μM BzATP in the absence or presence of 25 nM Bafilomycin-A1. **(C)** SOD1-G93A microglia were treated for 15 min with 300 μM BzATP or 3 mM ATP, in the absence or presence of 100 μM PD98059. Data represent mean ± SEM of *n* = 3 independent experiments. Statistical significance was calculated by ANOVA followed by *Post Hoc* Tukey’s test and referred to Ctrl (*p < 0.05, **p < 0.01, ***p < 0.001); to BzATP15’-treated cells or to ATP15’-treated cells (^#^*p* < 0.05).

As shown by immunoblotting in Figure 1A, SOD1-G93A microglia constitutively express augmented levels of LC3B-II with respect to WT microglia. Activation of P2X7 by the specific agonist BzATP (10–100–300 μM) increases LC3B-II protein content in a dose-dependent manner in SOD1-G93A microglia with maximal effect obtained at 300 μM (Figure 1B). The time-course experiment shows that the levels of LC3B-II are maximally induced after 15 min, with a consequent decline observed at 1–6 h (Figure 1B). Notably, the physiologic purinergic agonist ATP used at the mM concentrations required to activate P2X7 exerts similar effects (Figure 1C). LC3B-II induction by BzATP and ATP is decreased by the specific P2X7 antagonist A-804598 (10 μM; Figure 1D), thus indicating P2X7 involvement. Autophagy in SOD1-G93A microglia is moreover qualitatively assessed by immunofluorescence confocal analysis, by observing cells with accumulation of LC3 dots, because cells presenting a diffuse distribution of LC3 are generally described as non-autophagic, whereas cells with increased LC3 vacuoles are classified as autophagic (Tasdemir et al., 2008; Klionsky et al., 2016). Confirming the western blot data, we observed that 300 μM BzATP increases cytoplasmic LC3-positive dots in microglia with respect to control, and the effect is reduced by the P2X7 antagonist A-804598 (Figure 1E). To demonstrate the autophagic mechanism involved in LC3-II induction, next we show that LC3B-II up-regulation by BzATP and ATP is attenuated by wortmannin (WM; 100 nM), an inhibitor of the initial stage of conventional autophagy/phosphoinositide-3-kinase (Klionsky et al., 2016; Figure 2A). A correct way to define if a treatment augments a true autophagic flux is to determine the levels of LC3B-II in the presence of the lysosomal proteases inhibitor bafilomycin-A1 (Klionsky et al., 2016). While the increase in LC3-II by a treatment simply indicates the accumulation of autophagosomes without proving autophagic degradation, an enhancement of autophagy indeed occurs if LC3-II further accumulates in the presence of bafilomycinA1 (Mizushima and Yoshimori, 2007). Here we show that the addition of bafilomycin-A1 (25 nM) indeed further increases the up-regulation of LC3B-II induced by 300 μM BzATP with respect to control cells, consistently with an accelerated true autophagic flux (Figure 2B).

We have previously demonstrated that BzATP modulates ERK1/2 phosphorylation in SOD1-G93A primary microglia (Apolloni et al., 2013b). Because the MAPK activation pathway is a positive regulator of autophagy induction (Young et al., 2015) and previous data have reported that p-ERK1/2 is specifically involved in induction of LC3B-II expression by activated P2X7 in non-transgenic microglia (Takenouchi et al., 2009b), we have stimulated SOD1-G93A microglia with 300 μM BzATP or 3 mM ATP in the presence of the MEK pathway inhibitor PD98059 (100 μM) that *per se* does not affect basal LC3B-II content (data not shown). Although a partial and not statistically significant inhibition is observed on LC3B-II up-regulation by ATP, LC3B-II induction mediated by P2X7 receptor is not apparently dependent on ERK1/2 phosphorylation, not being significantly inhibited by PD98059 (Figure 2C).

### P2X7 Inhibits mTOR Phosphorylation and Modulates M2 Markers in SOD1-G93A Microglia

**Figure 3:**
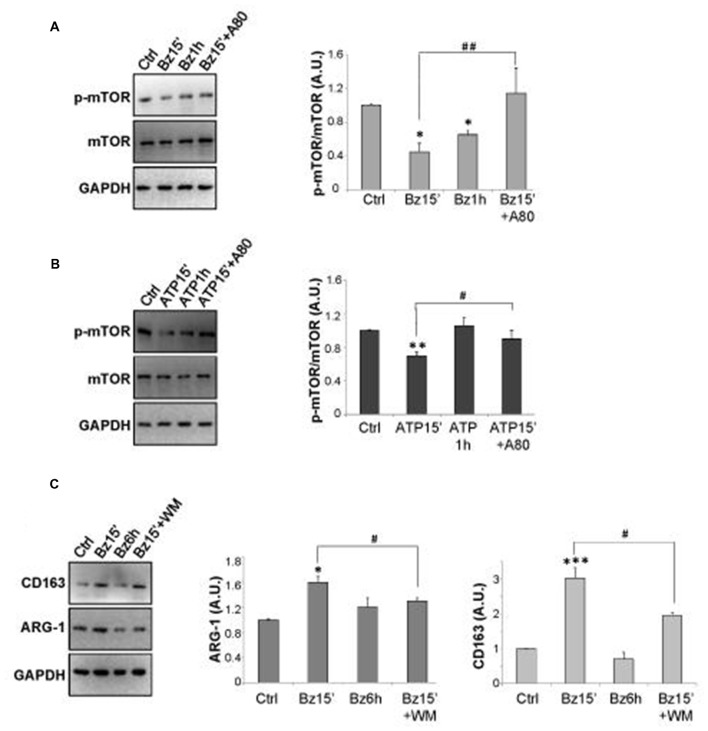
mTOR phosphorylation is regulated by P2X7 activation in SOD1-G93A microglia. SOD1-G93A microglia were treated with 300 μM BzATP **(A)** or 3 mM ATP **(B)** for 15 min or 1 h with or without 10 μM A-804598 and subjected to western blotting and immunoreactions with anti-p-mTOR and anti-mTOR or anti-GAPDH. SOD1-G93A cells exposed to 300 μM BzATP for 15 min with or without WM or 6 h were subjected to western blotting and immunoreactions with anti-Arginase-1 (ARG-1) **(C)** or anti-CD163 and anti-GAPDH. Data represent mean ± SEM of *n* = 3 independent experiments. Statistical significance was calculated by student’s *t*-test or Mann-Whitney rank sum test or ANOVA followed by *Post Hoc* Tukey’s test, and referred to Ctrl (*p < 0.05, **p < 0.01, ***p < 0.001); to BzATP15’- or ATP15’-treated cells (^#^*p* < 0.05, ^##^*p* < 0.01).

Inhibition of mTOR pathway is generally required for the induction of conventional autophagy (Klionsky et al., 2016). Thus, we have analyzed the effects of P2X7 activation on mTOR phosphorylation, showing by western blot analysis that 300 μM BzATP, or 3 mM ATP, is able to transiently decrease the protein content of p-mTOR/mTOR in SOD1-G93A microglia, with maximal inhibition observed after 15 min, as compared to control cells (Figures 3A,B). These effects are partially prevented by the P2X7 antagonist A-804598.

Microglia activation is known to occur by the alternating acquisition of different phenotypes concisely defined as M2 with anti-inflammatory properties, and M1 with pro-inflammatory features (Franco and Fernández-Suárez, 2015). Since the autophagic flux impacts on the M2/M1 microglia phenotypes (Su et al., 2016; Xia et al., 2016), we investigated the effects of BzATP stimulation on the levels of the M2 markers CD163 and ARG-1, in the absence or presence of WM. As shown in Figure 3C, 300 μM BzATP in 15 min increases the content of ARG-1 and CD163 proteins that returns approximately to basal levels in 6 h and is partially reverted by WM.

### Activation of P2X7 Affects SQSTM1/p62 Protein Content in SOD1-G93A Microglia and in Lumbar Spinal Cord of SOD1-G93A Mice

**Figure 4:**
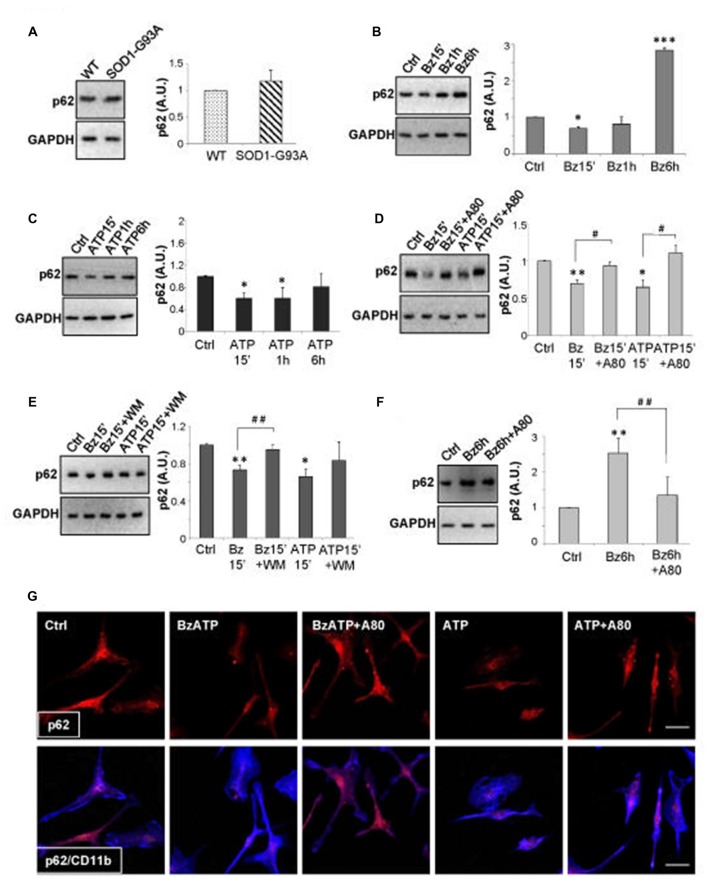
Activation of P2X7 affects SQSTM1/p62 levels in SOD1-G93A microglia. **(A)** Equal amounts of protein from SOD1-G93A and WT microglia were subjected to western blotting and immunoreactions with anti-SQSTM1/p62 and anti-GAPDH. SOD1-G93A microglia were exposed to 300 μM BzATP **(B)** or 3 mM ATP **(C)** for 15 min, 1 h or 6 h and equal amounts of total protein were subjected to western blotting and immunoreactions with anti-SQSTM1/p62 and anti-GAPDH. SOD1-G93A microglia were exposed to 300 μM BzATP or 3 mM ATP for 15 min with or without A-804598 10 μM **(D)** or WM 100 nM **(E)** and equal amounts of protein were subjected to western blotting and immunoreactions with anti-SQSTM1/p62 and anti-GAPDH. **(F)** SOD1-G93A microglia exposed to 300 μM BzATP for 6 h with or without 10 μM A-804598 were subjected to western blotting and immunoreactions with anti-SQSTM1/p62 and anti-GAPDH. **(G)** SOD1-G93A microglia treated for 15 min with 300 μM BzATP or 3 mM ATP in the absence or presence of 10 μM A-804598 were analyzed by means of fluorescence microscopy after staining with anti-SQSTM1/p62 (red) and CD11b (blue) (scale bar 50 μm). Data represent mean ± SEM of *n* = 3 independent experiments. Statistical significance was calculated by student’s *t*-test or ANOVA followed by *Post Hoc* Tukey’s test referred to Ctrl (*p < 0.05, **p < 0.01, ***p < 0.001); to BzATP15’-treated cells or ATP15’-treated cells (**D,E**; ^#^*p* < 0.05, ^##^*p* < 0.01), or BzATP6h-treated cells (**F**; ^##^*p* < 0.01).

A well-known substrate of autophagy is SQSTM1/p62 protein that can be degraded within the autolysosomes as an additional evidence of autophagic activity (Mizushima et al., 2010). To ascertain whether the increased LC3B-II levels induced by P2X7 activation in SOD1-G93A microglia represent a true increase in the autophagic flux, we have examined the content of SQSTM1/p62 protein. We have observed that while SOD1-G93A microglia contain levels of p62 comparable to WT microglia (Figure 4A), 300 μM BzATP (Figure 4B), or 3 mM ATP (Figure 4C), transiently decreases SQSTM1/p62 protein content in SOD1-G93A microglia with about 50% maximal inhibition obtained in 15 min (Figures 4B,C). A pre-incubation with the P2X7 antagonist A-804598 (Figure 4D) or with WM (Figure 4E), partially abolishes BzATP- and ATP-mediated SQSTM1/p62 decrease in SOD1-G93A microglia. As WM is also a class III phosphatidylinositol 3-kinase inhibitor, this result moreover indicates that P2X7-mediated increase in autophagic flux occurs via the phosphatidylinositol 3-kinase-mediated pathway. The reduction of SQSTM1/p62 by a short treatment with BzATP or ATP in SOD1-G93A microglia is confirmed also by double immunofluorescence with microglia specific marker anti-CD11b, as the intensity of the SQSTM1/p62 immunostaining is reduced after a short-term stimulation of P2X7 with respect to unstimulated cells, and the effect is reversed by the simultaneous presence of A-804598 (Figure 4G). Interestingly, a sustained activation of P2X7 by BzATP for 6 h markedly increases the levels of SQSTM1/p62 protein with respect to unstimulated cells, and this effect is abolished by the presence of A-804598 (Figure 4F), thus indicating that a prolonged P2X7 activation might cause an engulfment of the autophagic flux.

**Figure 5:**
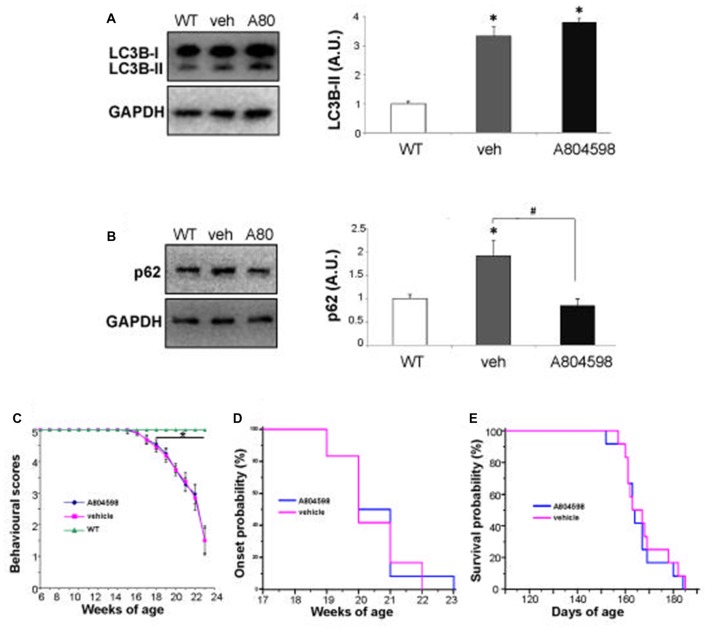
*In vivo* chronic treatment with P2X7 antagonist A-804598 decreases SQSTM1/p62 expression in lumbar spinal cord of SOD1-G93A mice. Equal amounts of protein from lumbar spinal cord lysates of WT mice (~5 months) and vehicle- or A-804598-treated SOD1-G93A mice at end stage (*n* = 4/group) were subjected to western blotting with anti-LC3B-II **(A)**, anti-SQSTM1/p62 **(B)** and anti-GAPDH. **(C)** Behavioral scores are not affected by A-804598 treatment (*n* = 12) with respect to vehicle mice (*n* = 12). WT mice (*n* = 3). Data represent mean ± SEM. Statistical significance was calculated by student’s *t*-test or ANOVA followed by *Post Hoc* Tukey’s test referred to WT (**A–C**; *p < 0.05) or to vehicle mice (**B**; ^#^*p* < 0.05). **(D)** The disease onset of SOD1-G93A mice, as shown by Kaplan–Meier survival curves was not affected by A-804598. **(E)** A-804598-treated mice show no significant differences in median survival with respect to vehicle-treated SOD1-G93A, as shown by Kaplan–Meier survival curves (163.5 days for A-804598 vs. 163 days for vehicle).

It is known that the SQSTM1/p62 autophagy substrate accumulates, together with LC3B-II, in lumbar spinal cord of ALS mice during disease progression, when the autophagic flux is impaired (Zhang et al., 2011). We have therefore measured the level of LC3B-II and SQSTM1/p62 proteins in lumbar spinal cord of SOD1-G93A mice after having pharmacologically inhibited *in vivo* the P2X7 receptor by a chronic treatment in female SOD1-G93A mice with the blood-brain permeant A-804598, demonstrated to reach brain concentrations in rodents after oral or i.p. doses (Able et al., 2011; Iwata et al., 2016), administered at 30 mg/Kg from pre-onset to end stage of disease. We find that while the protein levels of LC3B-II (Figure 5A) and SQSTM1/p62 (Figure 5B) are confirmed to be both increased at end stage in vehicle-treated SOD1-G93A mice with respect to WT, in A-804598-treated ALS mice with respect to vehicle, the LC3B-II protein content appears unmodified, while SQSTM1/p62 is inhibited to basal levels (Figures 5A,B). As shown, neither behavioral scores (Figure 5C), disease onset (Figure 5D), nor survival (Figure 5E) are however affected by A-804598 when administered in female SOD1-G93A mice.

## Discussion

**Figure 6:**
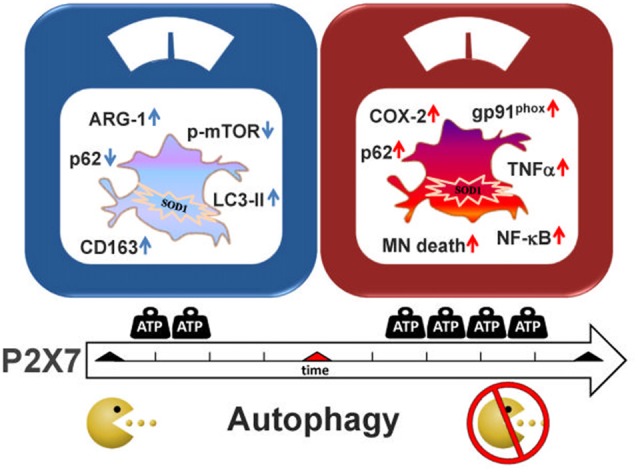
Schematic representation of P2X7 actions in SOD1-G93A microglia. Treatment of SOD1-G93A primary microglia with P2X7 agonists BzATP or ATP modulates inflammatory and autophagic markers. After a short stimulation (left panel), P2X7 induces an increase in M2 markers (ARG-1 and CD163) concomitantly with the stimulation of the autophagic process by augmenting LC3-II and decreasing p62 and p-MTOR levels. Conversely, a sustained activation with P2X7 agonists (right panel), causes inhibition of the autophagic flux with up-regulation of p62 that is parallel to previous results showing an increase of pro-inflammatory M1 markers gp91^phox^ (Apolloni et al., 2016b), cyclooxygenase-2 (COX-2; D’Ambrosi et al., 2009), and moreover nuclear factor kappa-B (NF-κB; Parisi et al., 2016), tumor necrosis factor alpha (TNF-α) and motor neurons (MN) death (D’Ambrosi et al., 2009).

Recent work has provided strong indications not only that autophagy plays a key role in ALS, but also that P2X7 receptor contributes to the neuroinflammatory pathway of ALS on one hand, and to the autophagic flux of microglia, on the other. The aim of this work was thus to investigate if P2X7 activation participates to ALS pathogenesis by directly modulating autophagic mechanisms in microglia, in other words to establish the occurrence of a P2X7-neuroinflammation-autophagy axis in ALS-microglia. We demonstrate that the modulation of the autophagic flux appears as a novel mechanism by which P2X7 contributes to activate SOD1-G93A microglia and to interfere with ALS features (Figure 6). In detail, we have illustrated here that a short time window of P2X7 stimulation by purinergic agonists augments LC3-II via the mTOR pathway and decreases SQSTM1/p62 levels, concurrently with an up-regulation of the M2 markers CD163 and ARG-1 in SOD1-G93A primary microglia. On the contrary, a sustained activation of P2X7 leads to an impairment of the autophagic flux with increased SQSTM1/p62 protein content. This might occur concomitantly with the previously characterized polarization of SOD1-G93A microglia toward the M1 phenotype exemplified by gp91^phox^, COX-2, TNF-α and NF-κB activation, and toxicity towards MN (D’Ambrosi et al., 2009; Apolloni et al., 2016b; Parisi et al., 2016). Although as yet unaware of the molecular mechanisms linking the decrease in autophagy to the insurgence of pro-inflammatory actions sustained by P2X7, our results are in line with the involvement of autophagy in the inflammatory response (Levine et al., 2011; Apolloni et al., 2016a). Indeed, when autophagy is stimulated by LPS in microglia, the expression of inducible nitric oxide synthase is suppressed (Han et al., 2013). Conversely, when the autophagic flux is inhibited, the M1 phenotype is induced with higher expression of TNF-α, inducible nitric oxide synthase and COX-2 mediated by the NF-κB pathway, and M2 genes are instead inhibited with down-regulation of ARG-1, BDNF and IL-10 (Xia et al., 2016). In both ALS patients and transgenic mice, it is well known that microglia switches from a beneficial M2 phenotype occurring during the early phases of disease, to a detrimental M1 phenotype that is more preponderant when the disease accelerates and motor neuron injury escalates (Henkel et al., 2009; Beers et al., 2011; Zhao et al., 2013; Hooten et al., 2015). These and our findings would thus suggest a parallelism between M2 inflammatory state and activation of autophagy, as well as M1 inflammatory condition and inhibition of autophagy, both consistently with the dual role played by P2X7 in ALS pathogenesis.

Moreover, we suggest that the short P2X7-dependent activation of autophagy demonstrated *in vitro* in SOD1-G93A microglia could be related to the beneficial role of P2X7 activation occurring during the early phase of the disease, when genetic ablation of P2X7 elicits detrimental consequences on ALS phenotype (Apolloni et al., 2013a). The persistent stimulation of P2X7 causing autophagic flux inhibition in SOD1-G93A microglia *in vitro* is instead perhaps resembling what occurs during the symptomatic phase of the disease, when the pharmacological inhibition of P2X7 exerts beneficial effects on ALS progression (Apolloni et al., 2014; Sluyter et al., 2017).

The initial stimulation of autophagic flux found in SOD1-G93A microglia by P2X7 activation with inhibition of mTOR phosphorylation differs from what reported in non-transgenic microglia where P2X7 signaling has been reported to negatively regulate autophagic flux and stimulate the phosphorylation of mTOR (Takenouchi et al., 2009b). The induction of microglial autophagy is instead in line with data obtained in dystrophic muscle cells, where P2X7 indeed increases autophagy (Young et al., 2015), thus indicating a phenotype-specific purinergic-mediated autophagy in ALS. In addition, we have demonstrated that LC3-II induction in SOD1-G93A microglia is apparently independent from ERK1/2 activation, again in line with what reported in muscle cells, where MAP kinases play only a marginal role in ATP-induced autophagy (Young et al., 2015). This is again different from what reported in non-transgenic microglia (Takenouchi et al., 2009b), where inhibition of autophagy is instead dependent on the MAPK pathway. By emphasizing the divergence between non-transgenic and SOD1-G93A microglia, these results however reinforce the synergism between autophagy and inflammation in P2X7-activated microglia.

Finally, in this article we have shown that inhibition of P2X7 through the antagonist A-804598 in SOD1-G93A mice suppresses SQSTM1/p62 up-regulation in lumbar spinal cord, thus confirming P2X7 as an *in vivo* modulator of the ALS pathological mechanism of autophagy, although further experiments might contribute to identify the cell phenotypes that are more responsible for altered autophagy. Survival, behavioral scores and disease onset are however not affected by A-804598 when administered in female SOD1-G93A mice at the time and dose adopted in our work. This suggests that inhibition of SQSTM1/p62 overexpression is perhaps necessary, but not sufficient, to ameliorate ALS disease. Given the various controversial male vs. female differences encountered on the effects of P2X7 antagonists or genetic ablation (Sluyter et al., 2017), we cannot exclude that further dose- and sex-dependent preclinical studies might provide different results.

Ectonucleotidase activities are responsible for the degradation of extracellular ATP, and SOD1-G93A microglia have a reduced extracellular ATP hydrolyzing activity with respect to non-transgenic microglia (D’Ambrosi et al., 2009; Butovsky et al., 2012; Volonté et al., 2016). This would imply that higher extracellular ATP concentrations might be present *in vivo* in the proximity of SOD1-G93A damaged MN to activate P2X7 on neighboring cells. While an initial activation of P2X7 might ensure a positive impact on microglia by stimulating autophagy, a persistent engagement of the receptor might in turn inhibit autophagy. These results thus extend to autophagy the dual role previously suggested for P2X7 in the SOD1-G93A model (Volonté et al., 2016). In order to further validate the *in vitro* and *in vivo* results about P2X7-dependent modulation of autophagy, because previous studies suggested that SOD1-G93A microglia has a regional heterogeneity during disease progression (Nikodemova et al., 2014), future experiments will investigate the actions of P2X7 also in primary microglia from SOD1-G93A spinal cord. Moreover, because WT-SOD1 overexpression in mice *per se* causes mitochondrial vacuolization, axonal degeneration and premature motor neuron loss in mice (Jaarsma et al., 2000), we will also study how this condition might for itself influence the effects of P2X7 in microglia.

In conclusion, our results extend the role of P2X7 as a means for interfering with autophagy in SOD1-G93A microglia, and a new mechanism of intervention against autophagic dysfunction in ALS pathogenesis.

## Author Contributions

PF, SAm and SAp performed experiments, collected and analyzed data. SAp and CV designed the experiments and wrote the manuscript. All authors approved the manuscript for publication.

## Conflict of Interest Statement

The authors declare that the research was conducted in the absence of any commercial or financial relationships that could be construed as a potential conflict of interest.

## Abbreviations

ALS: Amyotrophic Lateral Sclerosis
ARG-1: arginase-1
BzATP: 2′-3′-O-(benzoyl-benzoyl) ATP
COX-2: cyclooxygenase-2
LC3-II: microtubule-associated protein 1 light chain 3-II
MAPK: mitogen-activated protein kinase
NF-κB: nuclear factor kappa-B
NOX2: NADPH oxidase 2
SOD1: superoxide dismutase 1
TNF-α: tumor necrosis factor alpha
WM: wortmannin
WT: wild-type.
